# A new rhesus macaque assembly and annotation for next-generation sequencing analyses

**DOI:** 10.1186/1745-6150-9-20

**Published:** 2014-10-14

**Authors:** Aleksey V Zimin, Adam S Cornish, Mnirnal D Maudhoo, Robert M Gibbs, Xiongfei Zhang, Sanjit Pandey, Daniel T Meehan, Kristin Wipfler, Steven E Bosinger, Zachary P Johnson, Gregory K Tharp, Guillaume Marçais, Michael Roberts, Betsy Ferguson, Howard S Fox, Todd Treangen, Steven L Salzberg, James A Yorke, Robert B Norgren,

**Affiliations:** 1 Institute for Physical Science and Technology, University of Maryland, College Park, Maryland 20742, USA; 2 Department of Genetics, Cell Biology and Anatomy, University of Nebraska Medical Center, Omaha, Nebraska 68198, USA; 3 Non-Human Primate Genomics Core, Yerkes National Primate Research Center, Robert W. Woodruff Health Sciences Center, Emory University, Atlanta, Georgia 30322, USA; 4 Division of Neurosciences, Primate Genetics Program, Oregon National Primate Research Center, Oregon Health & Sciences University, Beaverton, Oregon 97006, USA; 5 Department of Pharmacology and Experimental Neuroscience, University of Nebraska Medical Center, Omaha, Nebraska 68198, USA; 6 Center for Computational Biology and Department of Biomedical Engineering, Johns Hopkins University School of Medicine, Baltimore, Maryland 21205, USA; 7 Current affiliation: National Biodefense Analysis and Countermeasures Center, Frederick, MD 21702, USA

**Keywords:** *Macaca mulatta*, Rhesus macaque, Genome, Assembly, Annotation, Transcriptome, Next-generation sequencing

## Abstract

**Background:**

The rhesus macaque (*Macaca mulatta*) is a key species for advancing biomedical research. Like all draft mammalian genomes, the draft rhesus assembly (rheMac2) has gaps, sequencing errors and misassemblies that have prevented automated annotation pipelines from functioning correctly. Another rhesus macaque assembly, CR_1.0, is also available but is substantially more fragmented than rheMac2 with smaller contigs and scaffolds. Annotations for these two assemblies are limited in completeness and accuracy. High quality assembly and annotation files are required for a wide range of studies including expression, genetic and evolutionary analyses.

**Results:**

We report a new *de novo* assembly of the rhesus macaque genome (MacaM) that incorporates both the original Sanger sequences used to assemble rheMac2 and new Illumina sequences from the same animal. MacaM has a weighted average (N50) contig size of 64 kilobases, more than twice the size of the rheMac2 assembly and almost five times the size of the CR_1.0 assembly. The MacaM chromosome assembly incorporates information from previously unutilized mapping data and preliminary annotation of scaffolds. Independent assessment of the assemblies using Ion Torrent read alignments indicates that MacaM is more complete and accurate than rheMac2 and CR_1.0. We assembled messenger RNA sequences from several rhesus tissues into transcripts which allowed us to identify a total of 11,712 complete proteins representing 9,524 distinct genes. Using a combination of our assembled rhesus macaque transcripts and human transcripts, we annotated 18,757 transcripts and 16,050 genes with complete coding sequences in the MacaM assembly. Further, we demonstrate that the new annotations provide greatly improved accuracy as compared to the current annotations of rheMac2. Finally, we show that the MacaM genome provides an accurate resource for alignment of reads produced by RNA sequence expression studies.

**Conclusions:**

The MacaM assembly and annotation files provide a substantially more complete and accurate representation of the rhesus macaque genome than rheMac2 or CR_1.0 and will serve as an important resource for investigators conducting next-generation sequencing studies with nonhuman primates.

**Reviewers:**

This article was reviewed by Dr. Lutz Walter, Dr. Soojin Yi and Dr. Kateryna Makova.

## Background

Rhesus macaques (*Macaca mulatta*) already play an important role in biomedical research because their anatomy and physiology are similar to humans. However, the full potential of these animals as models for preclinical research can only be realized with a relatively complete and accurate rhesus macaque reference genome.

To take advantage of the powerful and inexpensive next-generation sequencing (NGS) technology, a high quality assembly (chromosome file) and annotation (GTF or GFF files) are necessary to serve as a reference. Short NGS reads are aligned against chromosomes; the annotation file is used to determine to which genes these reads map. For example, in mRNA-seq analysis, mRNA reads are aligned against the reference chromosomes. The GTF file is used to determine which exons of which genes are expressed. If the genome used as a reference is incomplete or incorrect, then mRNA-seq analysis will be impaired.

The publication of the draft Indian-origin rhesus macaque assembly rheMac2 [1] was an important landmark in nonhuman primate (NHP) genomics. However, rheMac2 contains many gaps [1] and some sequencing errors [2,3]. Further, some scaffolds were misassembled [3,4] while others were assigned to the wrong positions on chromosomes [3-5]. There have been a number of attempts to annotate rheMac2 including efforts by NCBI, Ensembl and others [6,7]. However, it is not possible to confidently and correctly annotate a gene in an assembly with missing, wrong or misassembled sequence. It is important to note that even a single error in the assembly of a gene, for example a frameshift indel in a coding sequence, can produce an incorrect annotation [3].

Since the publication of rheMac2, another rhesus macaque genome was produced from a Chinese-origin animal: CR_1.0 [8] (referred to as rheMac3 at the University of California at Santa Cruz Genome Browser). Whole genome shotgun sequencing was performed on the Illumina platform generating 142 billion bases of sequence data. Scaffolds were assembled with SOAPdenovo [8]. These scaffolds were assigned to chromosomes based partly on rheMac2 and partly on human chromosome synteny [8]. Hence, this was not a completely new assembly as errors in scaffold assignment to chromosomes in rheMac2 were propagated to the CR_1.0 assembly. Further, the CR_1.0 contig N50 was much lower than for rheMac2 indicating a more fragmented genome. Annotations for CR_1.0 are available in the form of a GFF file. Although Ensembl gene IDs are provided in this file, gene names and gene descriptions are not, limiting the use of these annotations for NGS.

We have produced a new rhesus genome (MacaM) with an assembly that is not dependent on rheMac2. Further, we provide an annotation in a form that can be immediately and productively used for NGS studies, i.e., a GTF file which provides meaningful gene names and gene descriptions for a significant portion of the rhesus macaque genome. We demonstrate that both the assembly and annotation of our new rhesus genome, MacaM, offer significant improvements over rheMac2 and CR_1.

## Methods

### Genomic DNA sequencing

We obtained genomic DNA from the reference rhesus macaque (animal 17573) [1] and performed whole genome Illumina sequencing on a GAIIx instrument, yielding 107 billion bases of sequence data. We deposited these sequences in the Sequence Read Archive (SRA) under accessions [GenBank:SRX112027, GenBank:SRX113068, GenBank:SRX112904]. In addition, we used a human exome capture kit (Illumina TruSeq Exome Enrichment) to enrich exonic sequence from the reference rhesus macaque genomic DNA. Illumina HiSeq2000 sequencing of exonic fragments from this animal generated a total of 17.7 billion bases of data. We deposited these sequences in the SRA under accession [GenBank:SRX115899].

### Contig and scaffold assembly

We assembled the combined set of Sanger (approximately 6× coverage), Illumina whole genome shotgun (approximately 35× coverage) and exome reads using MaSuRCA (then MSR-CA) assembler version 1.8.3 [9]. We pre-screened and pre-trimmed the Sanger data with the standard set of vector and contaminant sequences used by the GenBank submission validation pipeline. The MaSuRCA assembler is based on the concept of super-read reduction whereby the high-coverage Illumina data is transformed into 3-4× coverage by much longer super-reads. This transformation is done by uniquely extending the Illumina reads using k-mers and then combining the reads that extend to the same sequence. We transformed the exome sequence data from the reference animal into a separate set of exome super-reads. We then used these exome super-reads along with Sanger and whole genome shotgun Illumina data in the assembly. The exome super-reads were marked as non-random and therefore were excluded from the contig coverage evaluation step that is designed to distinguish between unique and repeat contigs.

### Chromosome assembly steps

**Figure 1:**
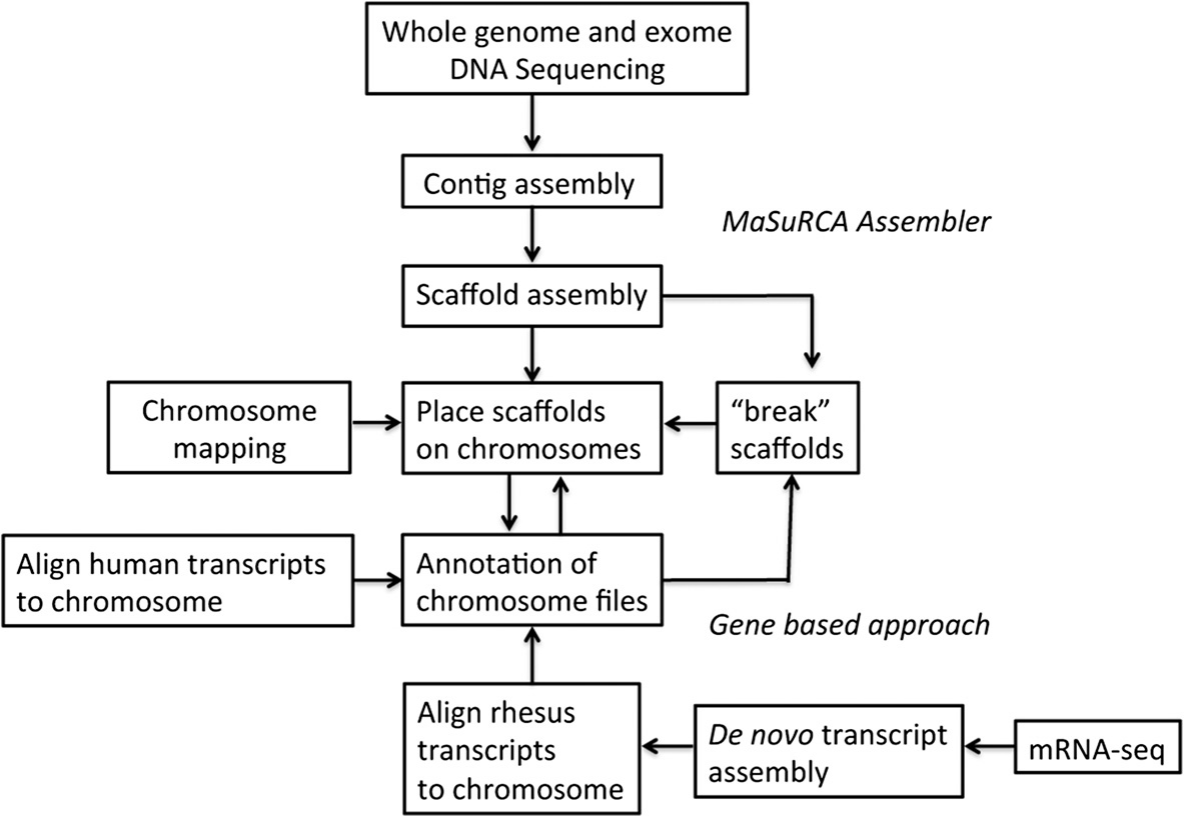
Flowchart illustrating procedures for assembly and annotation of the MacaM rhesus macaque genome.

A flowchart (Figure 1) illustrates the overall process of assembly and annotation.

We used BLAST + (version 2.2.25) for all BLASTn [10] alignments. We used default parameters for BLASTn alignments with the following exceptions:

–num_descriptions = 1; -num_alignments = 1; -max_target_seqs = 1.

1. We used BLASTn [10] to map exons from well-annotated human genes (Additional file 1) to scaffolds and re-ordered contigs so that, for protein coding orthologs, exons from each gene were in the correct order and orientation. This contiguity rule was used to enforce consistency whenever it was violated in subsequent steps.

2. There are several published reports of radiation hybrid mapping in rhesus macaques [11,12]. We used BLASTn to align markers identified in these studies with MaSuRCA scaffolds. We then used marker order information from the radiation hybrid studies to place scaffolds containing these markers in the correct order on chromosomes.

3. FISH mapping with human BACs has been used to identify syntenic blocks in rhesus macaques [5,13,14]. We cross-referenced these assignments with the locations of human genes within each block. We then used BLASTn exon ranges identified in step 1 to find the location of orthologous rhesus genes within the identified syntenic blocks. We placed scaffolds containing these genes not already placed from step 2 on chromosomes according to the published synteny blocks.

4. There were still some scaffolds unplaced after step 3 as the radiation hybrid and FISH markers do not cover all portions of the rhesus chromosomes. To identify orthologous regions, we split human chromosome sequences into segments of 10,000 bp and used MegaBLAST [15] to align these segments against unplaced scaffolds. We then placed these scaffolds within the syntenic blocks defined by steps 2 and 3 in human chromosome order. As a result, small inversions and translocations may not be correctly represented.

5. Manual curation was used to resolve inconsistencies among the different sources of information.

We developed a new chromosome nomenclature for the rhesus macaque (Table 1). Our goal was to designate chromosomes in accord with human and great ape nomenclature to facilitate comparison of rhesus macaque genes and chromosomes with these species. Chimpanzees, gorillas, and orangutans have the same general chromosomal structure as humans, with one notable exception. The human chromosome 2 appears to be the result of a fusion event that occurred during hominid evolution. Thus, both the great apes and rhesus macaques have two chromosomes that roughly correspond to the short and long arms of human chromosome 2. In the great apes, these two chromosomes are referred to as 2a and 2b. We adopted the same nomenclature for rhesus macaques to make comparisons between different primates easier.

**Table 1:** Rhesus chromosome nomenclature

**H**	**C**	**G**	**O**	**M**	**W**	**R**
1	1	1	1	1	1	1
2p+	2a	2a	2a	2a	15	13
2q-	2b	2b	2b	2b	9	12
3	3	3	3	3	3	2
4	4	4	4	4	4	5
5	5	5	5	5	5	6
6	6	6	6	6	6	4
7/21	7/21	7/21	7/21	7	2	3
8	8	8	8	8	8	8
9	9	9	9	9	14	15
10	10	10	10	10	10	9
11	11	11	11	11	11	14
12	12	12	12	12	12	11
13	13	13	13	13	16	17
14/15	14/15	14/15	14/15	14	7	7
20/22	20/22	20/22	20/22	15	13	10
16	16	16	16	16	20	20
17	17	17	17	17	17	16
18	18	18	18	18	18	18
19	19	19	19	19	19	19
X	X	X	X	X	X	X
Y	Y	Y	Y	Y	Y	Y

H = Human; C = Chimpanzee; G = Gorilla; O = Orangutan; M = MacaM; W = Wienberg et al. [16]; R = Rogers et al. [17].

We have deposited our new rhesus macaque assembly (MacaM_Assembly_v7) in NCBI’s BioProjects database under accession [GenBank:PRJNA214746].

### Chromosome assembly validation

We used genomic DNA from the reference rhesus macaque (animal 17573) to create a 400 bp library according to manufacturer’s instructions (Ion Torrent, Personal Genome Machine). We sequenced this library on an Ion 318 chip and deposited 1.5 billion bases of sequence in the SRA under accession [GenBank:SRR1216390]. To independently assess genome assembly, we aligned these Ion Torrent reads (which were not included in our assembly) against rheMac2, CR_1.0 and MacaM assemblies with TMAP 4.0 [18].

### RNA sequencing and transcript assembly

We extracted RNA from 11 samples using standard methods and performed sequencing with an Illumina Genome Analyzer IIx. We sequenced RNA from the cerebral cortex from 6 different animals at 76 bp, (single-end reads) and deposited the sequences in the SRA under accessions [GenBank:SRX099247, SRX101205, SRX101272, SRX101273, SRX101274 and SRX101275]. We sequenced RNA from the caudate nucleus of one animal at 76 bp (paired-end reads) and deposited the sequences at SRA under accession [GenBank:SRX103458]. We sequenced RNA from the caudate nucleus, cerebral cortex, thymus, and testis from a single rhesus macaque, 001T-NHP, at 76 bp for the caudate nucleus and at 100 bp for the other tissues (paired-end reads for all samples) and deposited the sequences in the SRA under the accessions [GenBank:SRX101672, SRX092157, SRX092159 and SRX092158], respectively.

To filter out genomic contamination, we aligned reads against human RefSeq mRNA transcripts using BLASTn [10]. For 76 bp reads, we filtered out sequences if they had an alignment length of 70 bp or less with human transcripts. For 100 bp reads, we filtered out sequences if they had an alignment length of 90 bp or less with human transcripts. For paired-end reads, we also removed a read if its mate was removed. We assembled filtered reads for each sample using Velvet-Oases [19,20]. We used K-mer values of 29 for the six samples with single reads and 31 for the remaining samples with paired-end reads. We set the coverage cutoff and expected coverage to ‘auto’. We set the minimum contig length to 200 bp. We used default parameters for Oases.

We obtained 369,197 *de novo* transcripts using the Velvet/Oases pipeline. We deposited transcripts in the NCBI’s Transcriptome Shotgun Assembly database under accessions [GenBank:JU319578 - JU351361; GenBank:JU470459 - JU497303; GenBank:JV043150 - JV077152; GenBank:JV451651 - JV728215] (Additional file 2). We also used reference-guided transcriptome assembly to identify rhesus transcripts. We performed spliced alignment of the rhesus RNA-seq reads to the MaSuRCA assembly using TopHat2 [21] (version 2.0.8b, default parameters). We provided the resulting BAM file of the read alignments as input to Cufflinks2 [22] (version 2.02, default parameters) for reference guided transcriptome assembly.

To identify rhesus orthologs of human genes, we used the BLASTx [23] program to align conceptual translations from the assembled rhesus transcripts against human reference proteins. We used the top hit in the annotation. We did not use a single set of cutoff values to identify orthologs. Instead, alignment lengths and percent similarity were manually inspected (see Annotation, procedure 1 for rationale). We identified a total of 11,712 full-length proteins representing 9,524 distinct genes with full length coding regions using the *de novo* and reference-guided methods described above.

### Annotation

We produced a GTF file (MacaM_Annotation_v7.6.8, Additional file 3) that serves as our annotation of the MacaM assembly. We used the following procedures to generate this file:

1. We used sim4cc [24] and GMAP [25] to align transcripts (both rhesus macaque and human) against the MacaM assembly to identify exon boundaries. For sim4cc, we specified CDS ranges for the transcripts which allowed sim4cc to identify the ranges of CDS within exons. For GMAP, we determined CDS ranges by concatenating sequences from proposed exons and then used this transcript model as the query in a BLASTx [23] search against human proteins. We then used a custom script to calculate CDS ranges within the chromosome files.

To determine whether gene models produced by these automated annotators should be accepted or rejected for our final annotation, we parsed the GTF files from both sim4cc [24] and GMAP [25] with the gffread utility from the Cufflinks package [26] to construct protein sequences. We aligned these protein sequences with human protein sequences using the Emboss Needle program (which implements the Needleman-Wunsch algorithm [27]) to extract identity, similarity and gap values for each rhesus protein model. Our expectation was that most rhesus macaque proteins and their human orthologs would have a protein length difference of less than 5 amino acids and a protein similarity of greater than 92%. If no gene model was found which met these parameters for a given gene, values were manually inspected. Lower values were accepted for genes known to be poorly conserved across species, e.g., reproductive and immune system genes but were rejected for genes known to be highly conserved across species, e.g. neural synapse genes.

2. We identified some rhesus macaque exons, missed by sim4cc and GMAP, by aligning the orthologous human exon with the MacaM assembly using BLASTn [10].

3. We manually annotated some rhesus macaque exons after inspection of mRNA-seq alignments against the MacaM assembly with the Integrative Genome Viewer (IGV) [28].

4. We used synteny between human and rhesus macaque genomes to resolve difficult gene structures and paralogs.

5. If the 3′ end of the apparent penultimate exon and terminal exons of a gene were non-coding and were within 1 kb of each other, we included the intervening sequence to create a new terminal exon. In addition, we aligned human terminal exons against the MacaM assembly to extend rhesus terminal exon annotations through the 3′ UTR to the end of the exon. These steps were necessary because sim4cc and GMAP sometimes failed to annotate the terminal exon completely, presumably due to the lower level of conservation in the 3′ UTR.

6. We used several approaches to identify and correct errors in the GTF file that serves as our annotation of the new rhesus genome (MacaM_Annotation_7.6, Additional file 3). These included Eval [29] and gffread from the Cufflinks2 software packages [22]. We used custom scripts to ensure that every CDS range had a corresponding exon range and to remove duplicate transcripts. To correct the identified errors, we used a combination of custom scripts and manual editing.

We were able to identify complete protein models for 16,052 rhesus genes (Additional file 3) from a human gene target list of 19,063 named protein-coding genes (Additional file 1).

### Protein comparison

We downloaded the most recent human assembly (GRCh38) and GFF3 annotation from NCBI on February 6, 2014. To obtain a list of genes that contained only a single isoform, we filtered the GFF3 file so that only Gene IDs linked to a single RefSeq mRNA accession were retained. This procedure resulted in a list of 11,148 Gene IDs. We downloaded the most recent rhesus assembly, rheMac2, and the GFF3 annotation from NCBI on February 6, 2014 [30]. We then created a list of genes that was common to GRCh38, rheMac2, MacaM by determining if the gene name from the GRCh38 (human) single isoform list was also present in the NCBI annotation of rheMac2 and our new annotation of the MacaM assembly. We used the Cufflinks2 [22] tool, gffread, to obtain protein sequences for each of these genes in the two rhesus genomes. We then aligned the rheMac2/NCBI and MacaM proteins against their human protein orthologs using the EMBOSS [31] global alignment tool, needle v.6.3.1. Once the alignments were done, we used custom scripts to compile the results into a summary table (Additional file 4). We used this table to calculate mean values for Identity, Similarity and Gaps.

We previously identified a gene that was misassembled in rheMac2 – the Src homology 2 domain containing E (SHE) gene [3]. To compare models of the SHE gene from different assemblies and annotations, we attempted to find the proteins with this designation from several rhesus macaque annotations. Using BLASTp [32] with the human SHE protein as a query, we found a protein annotated by NCBI in the rheMac2 assembly as gene name “LOC716722”, protein definition “PREDICTED: SH2 domain-containing adapter protein E-like [Macaca mulatta]” under the accession XP_002801853.1. Ensembl annotated the SHE gene in rheMac2 with gene identification ENSMMUG00000022980. We obtained the protein sequence associated with this gene model (ENSMMUT00000032345) for comparison with other rhesus macaque annotations. We accessed the RhesusBase database (http://www.rhesusbase.org/) on June 17, 2014 and searched under Gene Symbol for “SHE” and Gene Full Name for “Src homology 2 domain containing E”. In both cases the message returned was “No gene found!”. In an attempt to see if the SHE gene was annotated in the CR_1.0 genome, we downloaded the CR.pep.fa file, containing proteins derived from this genome from the GIGA database site (http://gigadb.org/dataset/100002) containing sequences and annotations related to CR_1.0 on June 17, 2014. In this file, we identified a protein with the ENSEMBL identifier ENSMMUP00000030263, with a partial match to the rhesus macaque SHE protein we identified in MacaM. We searched the ENBSEMBL website for “ENSMMUP00000030263” and received a message directing us to the rhesus macaque SHE gene. This protein was also submitted to NCBI under accession EHH15290.1 where it is described as “hypothetical protein EGK_01357, partial [Macaca mulatta]”. The various putative rhesus macaque SHE proteins were aligned against the human SHE protein (NP_001010846.1).

### RNA expression analysis

We aligned raw reads from three samples: testes, thymus and caudate nucleus of the brain to both the rheMac2 and the MacaM assemblies using TopHat [21] (version 2.0.8) with default parameters. We analyzed those alignment files using Cufflinks2 [22], specifically Cuffdiff, to generate normalized expression (FPKM) values. The samples from which these mRNA sequences are derived are described above in the section on RNA sequencing and transcript assembly (accessions GenBank:SRX092158, GenBank:SRX092159 and GenBank:SRX101672).

We also obtained 60 peripheral blood mononuclear cell (PBMC) samples from rhesus macaques in a social hierarchy experiment performed at the Yerkes National Primate Research Center. We individually subjected 10 macaques that were dominant in the social hierarchy and 10 subordinate macaques to a human intruder as a stressor. Whole blood was then collected at several time points using a BD CPT vacutainer which allow for the collection of PBMCs. RNA was purified from PBMCs using the RNeasy kit (QIAGEN, Valencia CA). We prepared libraries using standard TruSeq chemistry (Illumina Inc., San Diego CA) and sequenced them on an Illumina Hi-Seq 1000 as 2 × 100 base paired-end reads at the Yerkes NHP Genomics Core Laboratory (http://www.yerkes.emory.edu/nhp_genomics_core/). Sequences were deposited at NCBI under accessions [GenBank:SAMN02743270 - SAMN02743329]. We mapped reads with STAR [33] (version 2.3.0e) to both the rheMac2 and MacaM genomes, using the reference annotations as splice junction references. rheMac2 annotations were obtained from UCSC. We discarded un-annotated non-canonical splice junctions, non-unique mappings and discordant paired-end mappings. We performed transcript assembly, abundance estimates and differential expression analysis with Cufflinks2 (version 2.1.1) and Cuffdiff 2 [22]. We determined differentially expressed transcripts for pair-wise experimental group comparisons with an FDR-corrected p-value (q-value) <0.05. We compared differences in unique read counts and mapping percentages between rheMac2 and MacaM assemblies using a paired T-test.

### Statement of ethical approval

Materials used in these studies were from animal work performed under Institutional Animal Care and Use Committee approval from the University of Nebraska Medical Center, Oregon Health and Sciences University and Yerkes National Primate Research Center. Animal welfare was maintained by following NIH (Public Health Service, Office of Laboratory Animal Welfare) and USDA guidelines by trained veterinary staff and researchers under Association for Assessment and Accreditation of Laboratory Animal Care certification, insuring standards for housing, health care, nutrition, environmental enrichment and psychological well-being. These met or exceeded those set forth in the Guide for the Care and Use of Laboratory Animals from the National Research Council of the US National Academy of Sciences.

## Results

### Assembly of MacaM

We combined Sanger sequences from the reference rhesus macaque with newly generated Illumina whole genome and exome sequences from the same animal and created new *de novo* contig and scaffold assemblies using the MaSuRCA assembler [9]. Although there are many procedures for generating scaffolds, they all produce misassemblies with greater or lesser frequency [34-37]. To identify and correct misassembled scaffolds and assign scaffolds to chromosomes, we used independent mapping data and preliminary scaffold annotation. To properly assign genomic segments, we introduced 395 breaks in the assembled scaffolds. There were a total of 2312 scaffolds assigned to chromosomes.

To guide the splitting of misassembled scaffolds and placement of scaffolds on chromosomes, we used BLASTn [10] to align human exons with rhesus scaffolds, identified rhesus radiation hybrid markers [11,12] within scaffolds, and used rhesus FISH maps to identify areas of synteny as well as species differences in chromosome structure [5,13,14].

We term our new assembly: MacaM.

### Annotation of MacaM

We annotated 16,050 genes and 18,757 transcripts with full-length coding sequences in the MacaM genome (from a target list of 19,063 genes, Additional file 1). To accomplish this, we used rhesus macaque Illumina mRNA-sequences to assemble a total of 11,712 transcripts representing 9,524 distinct genes (Additional file 3) using *de novo* (Table 2) and reference-guided transcriptome assemblers. We used these rhesus transcripts, as well as human data, in our new annotation of the rhesus genome. We were not able to annotate six genes in the assembly due to mutations in the reference animal (Table 3). We were able to annotate several MHC class II genes as well as major histocompatibility complex, class I, F (MAMU-F). However, the more polymorphic and repetitive MHC class I genes are difficult to assemble and annotate. Additional targeted sequencing of the reference animal, such as has been done for other rhesus macaques [38,39], is necessary to provide a high-quality assembly and annotation for these genes. Given the importance of rhesus macaques for immunological studies, finished sequencing of the reference rhesus MHC class I genes would be beneficial. The new rhesus annotation (MacaM_Annotation_v7.6) is available as a GTF file (Additional file 3).

**Table 2:** **Assembly statistics for ***de novo***rhesus transcripts**

**Sample accession**	**Tissue**	**Read length**	**Single or paired**	**N50**	**Median**	**Mean within 1 SD**
SRX099247	Cerebral cortex	76	Single	1578	547	643
SRX101205	Cerebral cortex	76	Single	1552	530	624
SRX101272	Cerebral cortex	76	Single	1659	496	626
SRX101273	Cerebral cortex	76	Single	1656	484	596
SRX101274	Cerebral cortex	76	Single	2054	590	722
SRX101275	Cerebral cortex	76	Single	1646	466	585
SRX103458	Caudate nucleus	76	Paired	2651	824	952
SRX101672	Caudate nucleus	76	Paired	2831	1079	1128
SRX092157	Cerebral cortex	100	Paired	2322	829	924
SRX092159	Thymus	100	Paired	2731	1060	1121
SRX092158	Testis	100	Paired	1970	641	753

**Table 3:** Mutations in the reference rhesus macaque which interfere with annotation

**Chromosome**	**Location**	**Gene symbol**	**Mutation**
1	135383631	KLHDC9	stop-gain
1	169148554	ZBTB41	start-loss
3	50555959	ZXDC	stop-gain
9	18304595	PRUNE2	deletion
10	23470221	C10orf67	start-loss
15	274407	PRPF6	stop-gain

All mutations were in the heterozygous state.

Future updates to the assembly and annotation will be made available here: http://www.unmc.edu/rhesusgenechip/index.htm#NewRhesusGenome.

### Comparison of MacaM with rheMac2 and CR_1

The MacaM assembly has 2,721,371,100 bp of sequence placed on chromosomes with a N50 contig size of 64,032 bp, more than double the size of the original published assembly and five times the size of the CR_1.0 assembly (Table 4).

**Table 4:** Chromosome assembly statistics

**Assembly**	**# contigs**	**Total bp of contigs**	**Max contig length**	**Mean contig length**	**Contig N50**	**Scaffold N50**
					**(kb)**	**(kb)**
rheMac2	172351	2646263223	219335	15354	28	6760
CR_1.0	399581	2562947788	205919	6414	13	1707
MacaM	93402	2721371100	560771	29136	64	3534

Contig statistics based on contigs placed on chromosomes.

To independently assess the completeness and accuracy of the three rhesus macaque assemblies, we aligned Ion Torrent reads from the reference rhesus macaque against each assembly. These reads had not been used in any of the assemblies, including MacaM. We were able to align 93, 94 and 98% of these reads to the rheMac2, CR_1.0 and MacaM assemblies, respectively. This suggests that the MacaM assembly is the most complete and accurate of the three available rhesus macaque assemblies.

The original NCBI annotation (in GFF format) of the rheMac2 assembly contains 20,973 genes. However, only 11,265 of these have been assigned informative gene names. The rest have generic names, most commonly a “LOC” prefix followed by a number. The rhesus proteins derived from our annotation of the MacaM assembly are much more similar to their human orthologs than those derived from the NCBI annotations of rheMac2 (Table 5, Additional file 4).

**Table 5:** Comparison of rhesus proteins extracted from rheMac2 and MacaM annotation files with human orthologs

**Annotation**	**Identity**	**Similarity**	**Gaps**
rheMac2_N	90.93	92.28	41.86
MacaM	97.02	98.16	1.12

rheMac2_N: NCBI annotation of rheMac2.

MacaM: Our annotation of the new MacaM assembly.

Values equal the mean percent Identities, Similarities and Gaps when comparing rheMac2_N and MacaM with human orthologs.

**Figure 2:**
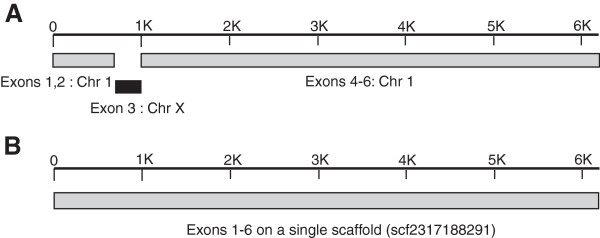
**Correction of rheMac2 SHE gene misassembly in MacaM. A**. rheMac2 genome. Exons 1, 2, 4, 5 and 6 of the Src homology 2 domain containing E. (SHE) gene are contained within scaffold NW_001108937.1. Exon 3 of this gene was assigned to scaffold NW_001218118.1. Scaffold NW_001108937.1 was correctly assigned to chromosome 1. However, scaffold NW_001218118.1 was mistakenly assigned to chromosome X. This resulted in an annotation of the rhesus SHE gene with missing sequence (corresponding to exon 3). Additional details on the misassembly of this gene in rheMac2 can be found in [3]. **B**. MacaM genome. All 6 exons of the SHE gene were found on scaffold 2317188291 of the MacaM assembly.

**Figure 3:**
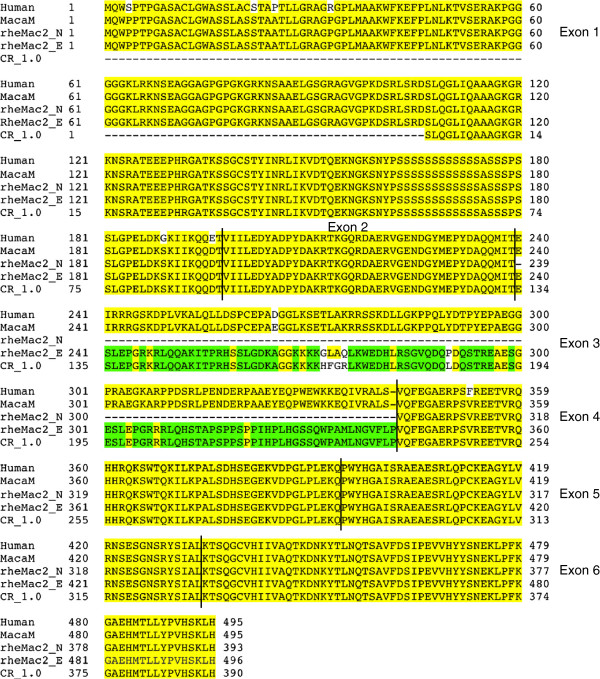
**Alignment of rhesus macaque SHE proteins from different annotations with human protein.** Human SHE protein accession: NP_001010846.1. MacaM: Protein derived from the MacaM rhesus macaque genome. rheMac2_N: Protein obtained from the NCBI annotation of rheMac2, accession. rheMac2_E: Protein obtained from the Ensembl annotation of rheMac2, accession ENSMMUT00000032345. CR_1.0: Protein obtained from the Chinese rhesus macaque genome produced by BGI [8]. Yellow highlighting indicates identical sequence in human and alternative rhesus macaque annotations with the exception of sequences that are only shared in rheMac2_E and CR_1.0 which are indicated by green highlighting. Exon boundaries are indicated by line separating amino acids.

We were able to annotate genes in MacaM whose exons had been split by misassemblies in rheMac2 [3] but which were contiguous in the MacaM assembly (Figure 2). We used this case (the SHE gene) to assess the annotations for rheMac2 and CR_1.0 (Figure 3). A search for SHE at the RhesusBase database indicated that no gene was found. We were able to identify proteins that appeared to correspond to the SHE protein in MacaM, rheMac2 (both NCBI and Ensembl annotations) and CR_1.0. We aligned these sequences against the human SHE protein sequence. The MacaM sequence was highly similar to the human SHE protein sequence. We found that a portion of the protein sequence encoded by exon 1 was missing in CR_1.0. The protein sequence encoded by exon 3 was missing from the NCBI annotation of rheMac2. For the Ensembl annotation of rheMac2 and CR_1.0, spurious sequence, presumably derived from intronic sequence, was substituted for the correct protein sequence encoded by exon 3. A correct protein sequence encoded by exon 3 was derived from MacaM because the scaffold containing exon 3 was correctly assembled which was not the case for rheMac2 [3] or, apparently, CR_1.0 (Figure 2). This demonstrates an important principle: better genome assemblies permit better annotations.

### mRNA expression studies with MacaM

When we compared mRNA-seq expression in three tissues (testis, thymus and caudate nucleus), we found that substantially more named genes were reported as expressed when the MacaM rhesus genome was used as compared to NCBI annotation of rheMac2 (Table 6).

**Table 6:** mRNA-seq expression comparison between rheMac2 and MacaM in four tissues

**Sample**	**# genes**	**# genes**	**Mean expression**	**Mean expression**
	**rheMac2_N**	**MacaM**	**rheMac2_N**	**MacaM**
Testis	5176	7587	45	47
Thymus	5177	7116	56	55
Caudate nucleus	4831	6726	51	52
Cerebral cortex	4963	6574	44	44

rheMac2_N: NCBI annotation of rheMac2.

MacaM: Our annotation of the new MacaM assembly.

All statistics were based on genes with an FPKM > =10.

**Figure 4:**
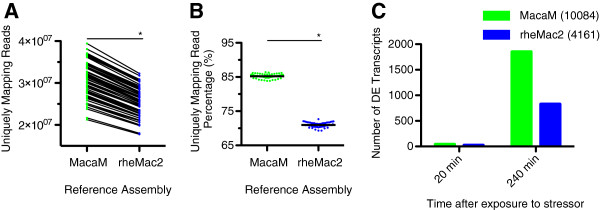
**mRNA expression validation.** We sequenced RNA from 60 rhesus macaque PBMC samples of differing ranks using Illumina paired end sequencing. After filtering, we mapped reads to either the MacaM (green symbols) or rheMac2 (blue symbols) assemblies using the STAR algorithm; we used CUFFLINKS to assign transcripts and determine differentially expressed genes (DEGs). **(A)** Number of uniquely mapping reads in individual RNA samples mapped using the MacaM and rheMac2 assemblies. Individual samples mapped by either assembly are joined by lines. **(B)** Percentage of total filtered reads that uniquely mapped to each assembly. **(C)** Number of DEGs that were identified using CUFFDIFF2.1 for dominant animals at two time points using the MacaM and rheMac2 genomes.

**Figure 5:**
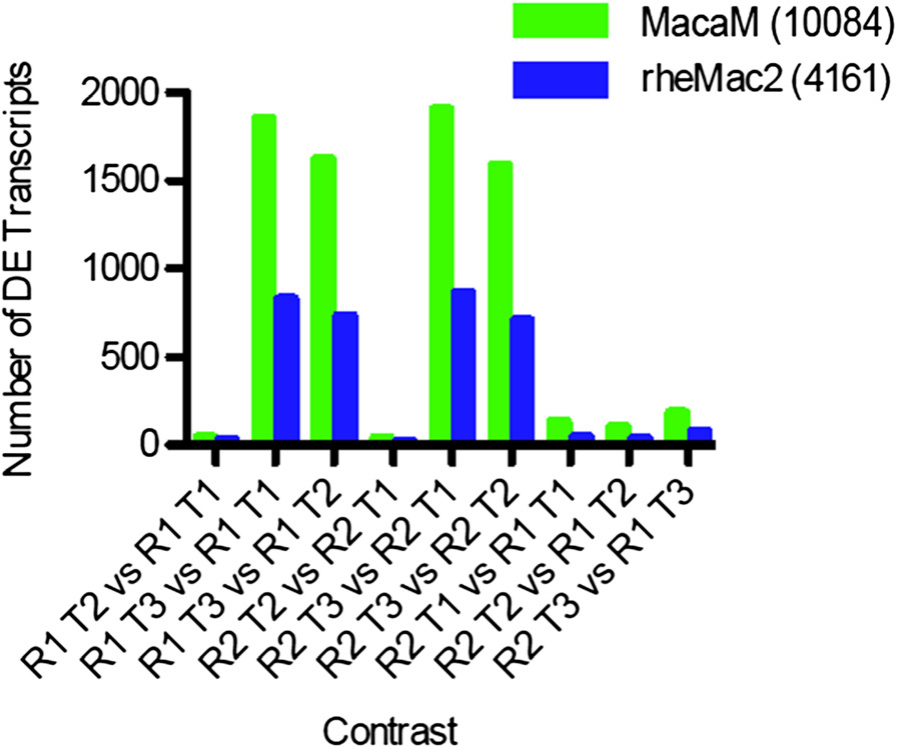
**Number of DEGs which were identified in an experiment analyzing social anxiety in rhesus macaques.** CUFFDIFF2.1 was used to identify DEGs with two Ranks (R1 = dominant; R2 = subordinate) and three time points (T1 = baseline; T2 = T1 + 20 minutes; T3 = T1 + 260 minutes). Human intruder intervention occurred immediately before T2, after T1.

To compare the performance of the rheMac2 and MacaM genomes on a ‘real-world’ mRNA-seq dataset, we examined the effects of an acute stressor on a background of chronic stress in social-housed female rhesus macaques. Social subordination in macaques is a natural stressor that produces distinct stress-related phenotypes and chronically stressed subordinate subjects [40]. We drew blood from both dominant and subordinate animals; following this, we exposed both to an acute stressor (human intruder paradigm [41]) and then drew blood at different time points after exposure. mRNA-seq read-mapping was significantly higher with MacaM as compared to rheMac2 (mean 3.1 × 10^7^ vs. 2.6 × 10^7^, p <0.0001) (Figure 4A). Similarly, mRNA-seq read-mapping percentages were significantly higher using the MacaM assembly than the rheMac2 assembly (mean 85.2% vs. 70.0%, p <0.0001 (Figure 4B). When we compared mRNA expression levels with one set of animals at two time points (Figure 4C), we detected many more Differentially Expressed Genes (DEGs) with the MacM genome than with rheMac2. We observed this same pattern in 8 additional comparisons (Figure 5).

## Discussion

There are several novel aspects to the approach we took to constructing a rhesus macaque genome as compared to previous efforts. First, we used both the original Sanger sequences and Illumina whole genome and exome sequences in the assembly. To accomplish this, we used a new assembler, MaSuRCA [9], to assemble contigs and scaffolds from this combination of sequences. The contigs we obtained had a much higher N50 than those obtained for rheMac2 or CR_1.0. Second, we used a preliminary annotation of scaffolds to identify and correct misassemblies and aid assignment of scaffolds to chromosomes. Third, we used mapping information for chromosome assignments that was not used for either the rheMac2 or CR_1.0 assemblies. Fourth, we used a combination of approaches, including both automated and manual curation, to provide a relatively complete, accurate and immediately useful annotation. NCBI has recently used our assembled transcripts to construct 3,392 rhesus macaque Reference Sequences (personal communication, Dr. David Webb, NCBI).

Users of genomes for NGS expression analysis require gene names and/or gene descriptions in the annotations to determine which genes are differentially expressed. Our MacaM GTF file provides gene names and gene descriptions for 16,050 genes with full-length coding sequences. The GenBank GFF file associated with rheMac2 provides meaningful gene names for 11,265 genes. Neither the RhesusBase annotation of rheMac2 nor the annotation provided for the CR_1.0 assembly contain gene names or gene descriptions. Although it is possible to link some of the Ensembl identifiers provided for RhesusBase and CR_1.0 to specific genes, this is a convoluted and uncertain process. It would be helpful if these annotation files were to include specific gene names so as to better allow a comparison of these annotations with others. In addition to being more complete, MacaM annotations were also more accurate than other available gene models for the rhesus macaque.

Typically, draft genome assemblies are produced by genome centers but are annotated by NCBI or EMBL using automated procedures. This practice has lead to annotation errors in a wide variety of species, including primates [3,42-44] because automated annotation procedures have difficulty coping with even small errors in assemblies. Our experience argues for integration of genome assembly and annotation. Preliminary annotation of scaffolds was critical for correcting some scaffold assembly errors and placing scaffolds in the correct order and orientation on chromosomes for MacaM. Thus, we argue for incorporation of annotation in the chromosome assembly pipeline.

MacaM could be used to improve other macaque genomes that were constructed at least in part with reference to rheMac2 [8,45]. However, given the similarities between Chinese origin rhesus macaques, cynomolgus macaques and the Indian rhesus macaque used as the reference animal for MacaM, it is likely that the MacaM genome could be used directly for alignments of Chinese origin rhesus macaque and cynomolgus macaque (*Macaca fascicularis*) NGS reads. It is likely that MacaM could be used as a reference genome for NGS studies with other macaque species such as *Macaca nemestrina*. It may be possible to use MacaM to aid in the assembly and annotation of other, somewhat more distantly related old world monkeys such as the baboon (*Papio anubis*) and sooty mangabey (*Cercocebus atys*).

## Conclusions

We have demonstrated that it is possible to significantly improve a draft mammalian genome with a modest amount of additional NGS, a new genome assembler [9], consideration of gene contiguity, full use of all mapping data and careful annotation. MacaM works well for NGS studies requiring a high quality reference genome. An early version of the MacaM genome has already been used for expression analysis in a rhesus macaque model of HIV [46].

## Reviewers’ comments

We thank Professor Lutz Walter at the German Primate Center and Dr. Soojin Yi at the Georgia Institute of Technology for their helpful comments. We have revised our manuscript accordingly.

### Reviewer #1: Professor Lutz Walter

Zimin and co-workers re-sequenced the Indian rhesus macaque individual that was used for the first genome assembly (rheMac2). They combined their Illumina sequences (whole genome as well as exome sequencing) with the original Sanger sequences (of rheMac2) to produce a new rhesus macaque genome assembly which they called MacaM. These efforts resulted in a weighted average contig size being twice as large as the original rheMac2 assembly and five times larger than the CR_1.0 (Chinese rhesus macaque) assembly and in correction of several misassemblies. Inclusion of Illumina RNA-seq data from different tissues (cerebral cortex, caudate nucleus, thymus, testis) improved gene annotations. Further, the authors used RNA-seq data from peripheral blood mononuclear cells (PBMCs) to demonstrate the superior accuracy and capability of the MacaM assembly to align reads from RNA-seq experiments.

The previous rhesus macaque genome assemblies and gene annotations urgently needed an update, which Robert Norgren’s group provided at the right time. Their data will help researchers to use the rhesus macaque genome much more efficiently.

Authors’ response: *We thank Professor Walter for his kind evaluation.*

Specific comments:

1) The PBMC samples are missing in the Material/Methods section.

Authors’ response: *We have now more fully explained our handling of the PBMC samples in the Methodssection (in the second paragraph under “RNA expression analysis”).*

2) The authors used samples from different animals such as cerebral cortex from 6 rhesus macaques or PBMC samples from 20 animals. So, they obviously obtained a lot of genetic variability data. However, they used information from different animals only for the analysis of transcript abundance (done only for PBMC samples). Why did the authors not present data on the cerebral cortex?

Authors’ response: *For the mRNA-seq expression comparison, we initially did not provide data on the cerebral cortex because we provided data from another region of the brain, the caudate nucleus, and wanted to emphasize results from widely varying tissues. However, we can understand the desire to include all four samples in this table. We have therefore added values for the cerebral cortex to Table *6*.*

Further, RNA-seq from 26 animals should contain a lot of SNP data. Is there also an increase in the ability to align SNPs on the MacaM assembly compared to rheMac2?

Authors’ response: *Professor Walter is correct that many SNPs could potentially be identified from the mRNA samples we obtained. However, using RNA-seq data for comparing MacaM with rheMac2 is problematical due to variability in transcript abundance. To interpret differences in candidate SNPs derived from alignments of mRNA reads against the two assemblies, one should ideally have a gold standard list of SNPs in a given tissue. Such a gold standard list has only recently become available in humans and is unfortunately not yet available in rhesus macaques. We agree that a comparison between rheMac2 and MacaM for mRNA-seq SNPs should be done as soon as a gold standard list of SNPs is established.*

3) The accompanying additional files indicate that MacaM does not contain non-coding RNA. Any plans to include this?

Authors’ response: *Yes, this is on our list of things to do. We expect to continue to update and improve both the MacaM assembly and annotation based on user input. Future updates to the assembly and annotation will be posted here: http://www.unmc.edu/rhesusgenechip/index.htm#NewRhesusGenome. We have also added this link to the text.*4) Statistical analyses of the data shown in Figures 4C and 5 are missing.

Authors’ response: *We did not provide a statistical comparison for the expression analysis of the different behavioral groups because a manuscript focused on this question is in preparation. For the current work, our focus was on the differences between the two assemblies. Figures *4*C and *5 *were provided to indicate how prevalent these differences are. Since Dr. Yi (reviewer 2) had a related concern, we have deleted the last paragraph in the Results section relating to the different behavioral groups.*

5) I particularly acknowledge the new rhesus macaque chromosome nomenclature presented in Table 1, as there have been confusing reports in the past and nomenclatures of chromosomes were published that clearly contradicted published data.

Authors’ response: *We appreciate Professor Walter’s comment on this issue.*

### Reviewer #2: Dr. Soojin Yi

In this manuscript the authors provide a new sequencing and assembly of a rhesus macaque using the animal used to generate the widely used RheMac1 assembly. Rhesus macaque is a promising nonhuman primate model species and the utility of an improved rhesus macaque genome assembly for biomedical and comparative studies is tremendous. This paper contains abundant merits and I look forward to using the new assembly for research and education in the near future. The obvious merits of this work include 1) combining Sanger and Illumina reads for assembly 2) validation of assembly with Ion Torrent reads 3) direct comparison of the efficiencies of different assemblies for differential gene expression studies. The RNA sequencing and transcript assembly strategy as well as annotation strategy are excellent.

Authors’ response: *We thank Dr. Yi for her generous evaluation of our work.*

I have a few technical comments most of which are for clarifications or details that will be helpful for future users.

p.2 and throughout the manuscript: the authors refer to the rheMac3 assembly as CR_1.0. It may help avoiding confusion if the authors used a more widely used term CR_1.0/rheMac3 (as in UCSC browser).

Authors’ response: *We agree with Dr. Yi that we should note that CR_1.0 is termed rheMac3 in the UCSC genome browser and have now done so in the text. However, this is a bit of a controversial issue as only the original submitter of an assembly is technically allowed to issue updates. Baylor may wish to update their assembly, rheMac2, and would normally refer to their update as rheMac3. Because BGI has termed their assembly rheMac3, there may be some confusion if/when Baylor attempts to name their next update. We named our assembly MacaM to avoid contributing to this confusion.*

p.5/Table 1: I would appreciate more details on the logic underlying the proposed new chromosome nomenclature.

Authors’ response: *Chimpanzees, gorillas, and orangutans have the same general chromosomal structure as humans, with one notable exception. The human chromosome 2 appears to be the result of a fusion event that occurred during hominid evolution. Thus, the great apes and rhesus macaques both have two chromosomes that roughly correspond to the short and long arms of human chromosome 2. In the great apes, these two chromosomes are referred to as 2a and 2b. We adopted the same nomenclature for rhesus macaques to make comparisons between different primates easier. We have added additional text to our manuscript to make this point more clearly.*

Adding chromosome nomenclature in other ape species may help the readers to see the merit of the proposed nomenclature system.

Authors’ response: *We have added chimpanzee, gorilla and orangutan to* Table 1 *as requested.*

p.6 identifying orthologs of human genes: as described currently it is not clear whether the reciprocal-best-blast hit strategy is used; the reciprocal step using human reference proteins against the rhesus macaque transcripts is not specified.

Authors’ response: *We did not use a reciprocal-best-blast hit strategy to identify orthologs of human genes.*

Also the cutoff values need to be given (e.g. e-value coverage similarity criteria).

Authors’ response: *A single set of cutoff values was not used because different types of genes evolve at different rates. Our rationale for this is similar to that used to determine whether sim4cc and GMAP gene models were correct. We have added text to the manuscript to make this clearer.*

p.7 p.9 discussions on protein sequence annotation and exon misassembly: The authors use the example of the Src homology 2 domain containing E (SHE) gene to demonstrate the utility of MacaM assembly and how different annotations differ in their qualities.

However I failed to find a mention of this gene until this specific section. It will be nice to see why this specific example has been selected.

Authors’ response: *We agree that our discussion of the misassembly of the SHE gene begins a bit abruptly. We selected this gene because we had previously identified it as misassembled in another paper *[3]*. We have added text to the Protein Comparison section of the Methods to make this clear.*

Did they perform a global comparison between MacaM versus rheMac2? If they did it will be nice to see a table of such results (beyond the Table 5 which simply indicates identity/similarity/gaps) something like the mismatch of exon numbers and so on would be useful) as well as the list of curated/improved gene sets. Even though this is an optional comment I feel that detailed discussion on such results would also be a nice addition to support the authors’ conclusion that the MacaM is a better assembly and thus offers a better annotation.

Authors’ response: *No, we did not do a global comparison of gene assembly between MacaM and rheMac2. We discovered a number of misassembled genes in rheMac2, described in *[3]*, in the course of attempting to annotate that assembly. I cannot say that we have found them all. Automation only takes one so far in annotation. To confirm that a given gene model is correct in one assembly and wrong in another takes a tremendous amount of manual effort, unfortunately.*

p.9 please provide the full name for the gene MAMU-F. People who are not directly working with MHC class genes are not likely to understand.

Authors’ response: *This was an oversight on our part. We thank Dr. Yi for pointing it out. We added the full gene name for MAMU-F to the text.*

p.9 Comparison of MacaM with rheMac2 and CR_1: I understand what the authors are saying but I would remove or edit the three sentences following (Table 4) as this discussion is not directly validated with the identification of misassemblies in rheMac2.

Authors’ response: *We have removed these three sentences, as suggested.*

p.10 mRNA expression studies with MacaM: the authors conclude that MacaM assembly provides biologically relevant information while rheMac2 doesn’t. I agree that the results described supports “MacaM assembly provides biologically relevant information” but I am not seeing the support for “while rheMac2 doesn’t”. Perhaps they authors can elaborate.

At any rate these new data sound very interesting and perhaps the authors are preparing to publish them independently. If the authors decide not to include this data in the current manuscript so that they can publish in another paper I feel it is justified.

Authors’ response: *Indeed, a manuscript focused on behavior is in preparation. We have deleted the last paragraph in the Results section relating to differences in the behavioral groups.*

p. 11 a possible future direction is to complement the evidence-based assembly as done here with a purely computational gene prediction. This way we may be able to identify genes that are not easy to annotate based upon transcript information alone (RNA-seq data) or those that are not homologous to human genes (ortholog approach and exome approach).

Authors’ response: *We acknowledge that our approach may miss genes present in rhesus macaques but not in humans. Gene prediction programs are improving. So, it may be possible that additional genes will be identified using purely computational methods.*

### Reviewer #3: Dr. Kateryna Makova

This reviewer provided no comments for publication.

## Abbreviations

NGS: Next-generation sequencing; SRA: Sequence read archive; IGV: Integrative genome viewer; PBMC: Peripheral blood mononuclear cell; SHE: Src homology 2 domain containing E.

## Competing interests

The authors declare that they have no competing interests.

## Authors’ contributions

RBN conceived and organized the overall project. RBN, JAY, SLS, SEB, ZPJ, BF and HSF supervised the project; AVZ supervised the development of MaSuRCA, performed the contig and scaffold assemblies and submitted the assembly to NCBI; ASC contributed to the annotation and expression analysis; MDM and XZ contributed to the chromosome assembly and annotation; DTM and KW performed Ion Torrent sequencing; RMG contributed to the annotation analyses; SP contributed to the annotation; SEB performed RNA-seq analysis; ZPJ performed the acute stress experiment and analyzed the associated expression data; GKT performed RNA-seq analysis; GM and MR contributed to MaSuRCA development; BF supervised mRNA extraction and Illumina sequencing; HSF supervised mRNA extractions; TT performed the reference-guided transcriptome assembly; SLS provided advice on bioinformatics issues; JAY supervised contig and scaffold assembly; RBN supervised chromosome assembly, annotation, and RNA-seq analysis. All authors read and approved the manuscript.

## Supplementary Material

Additional file 1 **Target Gene List.** List of human genes for which we attempted to obtain annotations of rhesus macaque orthologs. Includes human gene ID, gene symbol, gene description and whether we successfully annotated the rhesus ortholog (- = no, + = yes). Click here for file

Additional file 2 ***De novo *** **assembled transcripts.** Accessions and names of rhesus macaque transcripts we assembled using Velvet-Oases. Includes human gene ID, gene symbol, gene description and accessions of TSA records. Click here for file

Additional file 3 **MacaM_Annotation_v7.6.** Annotation of the MacaM assembly in GTF format. Click here for file

Additional file 4 **NCBI and MacaM protein comparison table.** Statistics comparing NCBI annotations of rheMac2 with our annotations of MacaM. Click here for file
